# LncRNA LINC01503 aggravates the progression of cervical cancer through sponging miR-342-3p to mediate FXYD3 expression

**DOI:** 10.1042/BSR20193371

**Published:** 2020-06-10

**Authors:** Xing Peng, Jinyu Gao, Chunyan Cai, Yumei Zhang

**Affiliations:** Department of Gynaecology, the Affiliated Huaian No.1 People’s Hospital of Nanjing Medical University, No.6 Beijing West Road, Huaian, 223300, Jiangsu, China

**Keywords:** FXYD3, miR-342-3p, LINC01503, CC

## Abstract

Cervical cancer (CC), an aggressive malignancy, has a high risk of relapse and death, mainly occurring in females. Accumulating investigations have confirmed the critical role of long noncoding RNAs (lncRNAs) in diverse cancers. LncRNA LINC01503 has been reported as an oncogene in several cancers. Nonetheless, its role and molecular mechanism in CC have not been explored. In the present study, we found that FXYD3 expression was considerably up-regulated in CC tissues and cells. Moreover, FXYD3 deficiency conspicuously hampered cell proliferation and migration while facilitated cell apoptosis in CC cells. Subsequently, molecular mechanism experiments implied that FXYD3 was a downstream target gene of miR-342-3p, and FXYD3 expression was reversely mediated by miR-342-3p. Moreover, we discovered that LINC01503 acted as the endogenous sponge for miR-342-3p. Besides, LINC01503 negatively regulated miR-342-3p expression and positively regulated FXYD3 expression in CC. Rescue assays revealed that LINC01503 depletion-induced repression on CC progression could be partly recovered by miR-342-3p inhibition, and then the co-transfection of sh-FXYD3#1 rescued this effect. Conclusively, LINC01503 aggravated CC progression through sponging miR-342-3p to mediate FXYD3 expression, providing promising therapeutic targets for CC patients.

## Introduction

Cervical cancer (CC) is the second malignant tumor that commonly occurs in females and results in the death associated with cancers [1,2]. Although tremendous efforts have been made to explore the pathogenesis of CC, the prognosis of CC patients remains largely poor [3,4]. In consequence, exploring the potential molecular mechanisms and searching new therapeutic methods are extremely urgent to improve the survival rate of CC patients.

Long noncoding RNAs (lncRNAs), over 200 nucleotides, are a type of transcripts with no ability to code proteins [5]. Recently, LINC01503 has been reported to promote tumorigenesis and progression of glioma by activating Wnt/β-catenin signaling [6]. LINC01503 is also overexpressed and plays oncogenic roles in esophageal squamous cell carcinoma [7]. In addition, LINC01503 facilitates cell proliferation and invasion in colorectal cancer through targeting miR-4492/FOXK1 axis [8]. Nonetheless, its role and molecular mechanism in CC are poorly understood.

FXYD domain containing ion transport regulator 3 (FXYD3), also named as mammary tumor 8, is a part of FXYD protein family and mainly distributed in cell membrane and cytoplasm [9]. It has been reported that FXYD3 functions as a regulator of sodium-potassium ATPase [10]. A former study has uncovered that the whole cell membrane protein is aberrantly expressed between tumor cells and normal cells, regulating cell metastasis, cell cycle, as well as the angiogenesis and development of tumors [11]. Previous investigations have revealed the aberrant expression of FXYD3 in diverse cancers, including prostate cancer [12], colorectal cancer [13], esophageal squamous carcinoma [14], breast cancer [15], pancreatic cancer [16], glioma [17] and lung cancer [18]. Moreover, FXYD3 has also been implied to be correlated with the prognosis of these cancers. However, the relationship between FXYD3 and progression of CC has not been investigated.

In the present study, we were devoted to studying the role and molecular mechanism of LINC01503 in CC. And it was discovered that LINC01503 aggravated CC progression through sponging miR-342-3p to mediate FXYD3 expression. This discovery provided promising biomarkers for CC treatment.

## Materials and methods

### Bioinformatics analysis

The expression pattern of FXYD3 in CC tissues and normal tissues was predicted using the 306 CESC (cervical squamous cell carcinoma and endocervical adenocarcinoma) tissue samples and 13 normal tissue samples in Gene Expression Profiling Interactive Analysis 2 (GEPIA2) database (http://gepia2.cancer-pku.cn/#analysis).

### Tissue samples

A total of 50 matched samples of CC tissues and adjacent non-cancerous tissues were collected for the present study between May 2014 and June 2019, under the ethical approval from the Ethics Committee of the Affiliated Huaian No.1 People’s Hospital of Nanjing Medical University. All patients had signed the written informed consents and none of them had received chemotherapy or radiotherapy prior to experiment. The tissue samples were instantly frozen at −80°C in liquid nitrogen after surgical resection for further analysis.

### Immunohistochemistry (IHC)

Fresh tissues from CC patients were fixed, dehydrated and embedded in paraffin. After cutting into 4 μm thick sections, IHC was carried out utilizing antibodies against FXYD3 (Abcam, Cambridge, MA, U.S.A.).

### Cell lines and culture

American Type Culture Collection (Manassas, VA, U.S.A.) commercially provided CC cells (SiHa, C-33A, HeLa and CaSki) and normal cervical epithelial cells (H8). Above cell lines were maintained in RPMI-1640 (Gibco, Life Technology, Carlsbad, CA, U.S.A.), supplemented with 10% fetal bovine serum and 1% penicillin/streptomycin in 5% CO_2_ at 37°C. The replacement of culture medium was conducted every third day.

### Quantitative real-time polymerase chain reaction (RT-qPCR)

The isolated total RNA was acquired from cultured HeLa and CaSki cells by use of TRIzol reagent (Invitrogen) on the basis of the manufacturer’s directions. The reverse-transcribed RNA was treated with PrimeScript™ RT Master Mix and SYBR® Premix Ex Taq™ II (Takara, Shiga prefecture, Japan) on the Bio-Rad CFX96 PCR System (Bio-Rad, CA, U.S.A.). For miRNA analysis, all procedures were in One™ miRNA RT-qPCR Detection Kit (GeneCopoeia, MD, U.S.A.). The final threshold cycle (*C*_T_) values of three repeats were calculated by 2^−ΔΔCt^ method and normalized to GAPDH or U6.

### Cell transfection assay

For silencing FXYD3 or LINC01503, the two specific shRNAs (sh-FXYD3#1/2 or sh-LINC01503#1/2) were bought from GeneCopoeia, along with the non-silencing shRNA (sh-NC) oligonucleotide as negative control. LINC01503 was amplified and inserted into the pcDNA3.1 vector (Invitrogen) for obtaining the vector pcDNA3.1/LINC01503. The empty pcDNA3.1 vector was taken as an internal reference. MiR-342-3p inhibitor, NC inhibitor, miR-342-3p mimics and NC mimics were designed and constructed by GenePharma (Shanghai, China). The cultured HeLa and CaSki cells in six-well culture plates were transfected with plasmids based on the guidebook of Lipofectamine 2000 (Invitrogen), and reaped after 48 h. The transfection efficiency was tested by RT-qPCR.

### Cell proliferation assays

The transfected HeLa and CaSki cells were placed into 96-well plates, and cultivated for 24 h at 37°C with 5% CO_2_. Cell viability was assessed over 4 days by use of the cell counting kit-8 (CCK-8; Dojindo, Kyushu, Japan). About 10 μl CCK-8 solution was added for 2 h. At length, a spectrophotometer was used to determine the absorption at 450 nm in each well. In addition, 2000 HeLa and CaSki cells were separately put into 6-well plates and routinely grown for 2 weeks, followed by fixation with formaldehyde and staining with 0.1% Crystal Violet. The colonies that contained over 50 cells were counted.

### Cell apoptosis assay

Cell apoptosis of HeLa and CaSki was evaluated by TUNEL staining method with in situ cell apoptosis detection kit (Shanghai, China). The fixed cells were centrifuged and washed twice with phosphate-buffered solution (PBS). Then, cell supernatant was removed; and double distilled water, bio-dNTP, terminal dexynucleotidyl transferase (TdT) and TdT buffer solution was added in sequence to mix with cells for half an hour at 37°C. Following centrifugation and washing, a fluorescence microscope was employed to observe cells after treatment of DAPI staining in the dark.

### Western blot

HeLa and CaSki cells were reaped and rinsed in PBS, followed by lysing in 100 µl of lysis buffer with protease inhibitor cocktail for 20 min on ice. The lysates were separated on the SDS-polyacrylamide gel and transferred into the PVDF membranes. Sealed with 5% defatted milk, samples were immunoblotted with the primary antibodies. The primary antibodies used were as below: anti-Bcl-2 (1:1000; ab32124, Abcam, Cambridge, MA, U.S.A.), anti-Bax (1:1000; ab32503, Abcam), anti-MMP2 (1:1000; ab92536, Abcam), anti-MMP9 (1:1000; ab76003, Abcam), anti-FXYD3 (1:1000; ab205534, Abcam), GAPDH (1:5000; ab9485, Abcam). Later, the samples were incubated with horseradish peroxidase-labeled secondary antibodies (1:5000; ab205718, Abcam), and the protein bands were directly viewed with an ECL detection reagent (Hercules, CA, U.S.A.), followed by analysis via ImageJ software.

### Cell migration assay

HeLa and CaSki cells were treated with different transfection plasmids for 48 h and then starved in culture medium without serum. Afterwards, the cells were seeded into the top chamber. Culture medium with 20% FBS was placed into the lower chamber. 24 h later, migrated cells were fixed in 4% paraformalin and dyed with 0.01% Crystal Violet. At last, number of cell migration was counted and observed by an inverted microscope.

### Luciferase reporter assay

To conduct luciferase reporter assay, HeLa and CaSki cell lines were planted into 24-well plates with the density of 400,000 cells per well prior to transfection. The synthetic wild-type or mutant reporter vectors of FXYD3 or LINC01503 (FXYD3-WT/Mut or LINC01503-WT/Mut) were acquired from GenePharma and co-transfected into HeLa and CaSki cells with miR-342-3p mimics or NC mimics using Lipofectamine 2000. The treated cells were reaped after 48 h and the luciferase intensity was assayed by the Dual-Luciferase Reporter system (Promega Corporation).

### RIP assay

RIP assay was performed in strict accordance with the the manufacturer’s protocol. A total of 1 × 10^7^ HeLa or CaSki cells were lysed in 100 μl of RIP lysis buffer containing protease and RNase inhibitors, following immunoprecipitation with antibodies of interest and magnetic beads all night at 4°C. Thereafter, the digested complex was purified and subjected to RT-qPCR for quantification.

### RNA pull-down assay

RNA pull-down assay was carried out with Biotin RNA Labeling Mix (Roche, Mannheim, Germany). Purified biotin-labeled miR-342-3p-WT, miR-342-3p-Mut or NC was cultivated with lysates from HeLa or CaSki cells for 1 h at room temperature, following addition with Dynabeads™ M-270 Streptavidin (Invitrogen) to extract bound RNAs. The final enrichment of LINC01503 or FXYD3 was tested by RT-qPCR.

### Statistical analysis

All experimental data from three replicates were showed as mean ± SD. Comparison between parameters were analyzed with a two-tailed unpaired Student’s *t*-test and one-way ANOVA. And Kaplan–Meier analysis was applied to analyze the association between FXYD3 expression and overall survival of CC patients. The threshold of significance was set as *P*<0.05.

## Results

### Overexpressed FXYD3 contributes to the progression of CC

**Figure 1:**
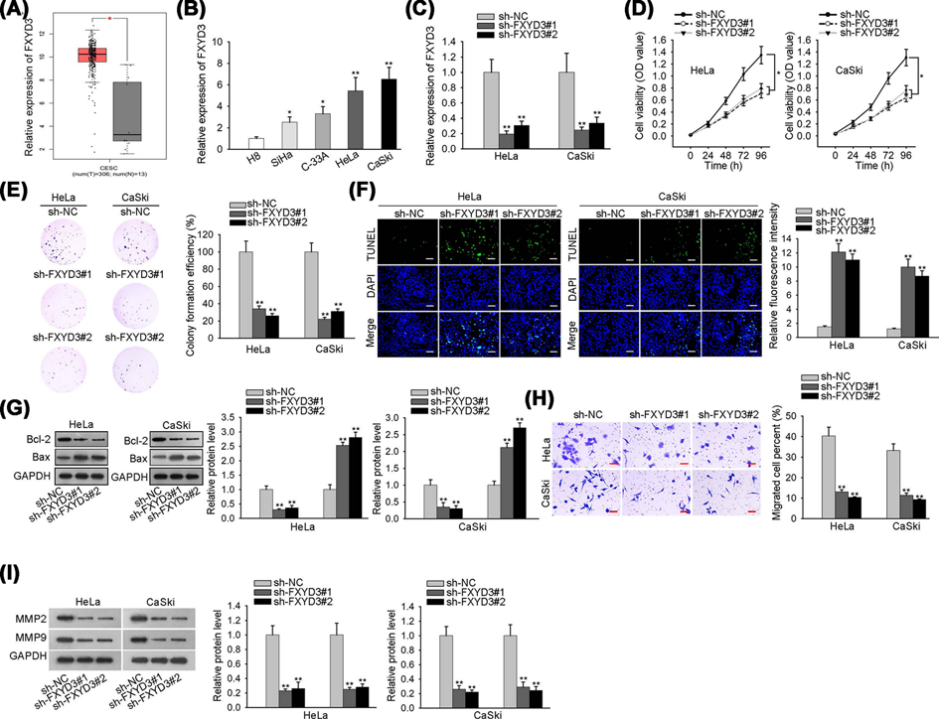
Overexpressed FXYD3 contributes to the progression of CC (**A**) FXYD3 expression level in CC tissues was identified by GEPIA2 database. (**B**) The expression level of FXYD3 in CC cell lines was assessed by RT-qPCR analysis. (**C**) RT-qPCR was applied to estimate the transfection efficiency of FXYD3 specific shRNAs. (**D** and **E**) CCK-8 and colony formation assays were employed to assess the effect of FXYD3 depletion on the proliferation ability of HeLa and CaSki cells. (**F**) TUNEL analysis was employed to measure the effect of FXYD3 silence on cell apoptosis; scale bar: 200 μm, magnification ×100. (**G**) The expression levels of apoptosis-related proteins (Bcl-2 and Bax) in HeLa and CaSki cells transfected with sh-FXYD3#1/2 or sh-NC was evaluated by Western blot analysis. (**H**) Transwell assay was utilized to detect the effect of FXYD3 depletion on cell migration; scale bar: 100 μm, magnification ×100. (**I**) Western blot analysis was used to test the expression levels of metastasis-related proteins (MMP2 and MMP9) in transfected cells; *P<0.05, **P<0.01.

According to online GEPIA2 database (http://gepia2.cancer-pku.cn/#analysis), we found that FXYD3 was at a high level in CC tissues (Figure 1A). Consistently, FXYD3 expression was significantly elevated in tissues obtained from CC patients when compared with matched peri-tumors (Supplementary Figure S1A,B). After applying Kaplan–Meier analysis, we observed that high expression of FXYD3 was associated with lower overall survival, suggesting that FXYD3 up-regulation could predict poor prognosis (Supplementary Figure S1C). Besides, FXYD3 expression in CC lines (SiHa, C-33A, HeLa and CaSki) and normal cervical epithelial cell line (H8) was detected by RT-qPCR, and the result unveiled that FXYD3 expression was remarkably overexpressed in CC cell lines (Figure 1B). To explore the biological role of FXYD3 in CC cells, FXYD3 was knocked down with shRNAs targeting FXYD3 (sh-FXYD3#1/2) (Figure 1C). Then, CCK-8 and colony formation assays were applied for assessing cell proliferation, and data indicated that FXYD3 deficiency resulted in a notable inhibition in the proliferation of HeLa and CaSki cells (Figure 1D,E). Later, TUNEL assay demonstrated that cell apoptosis was facilitated when HeLa and CaSki cells were transfected with sh-FXYD3#1/2 (Figure 1F). And Western blot analysis showed that Bcl-2 expression was decreased whereas Bax expression was increased in HeLa and CaSki cells which depleted FXYD3 (Figure 1G). In addition, we observed that FXYD3 downregulation repressed cell migratory capacity through transwell assay (Figure 1H). At last, the expression levels of metastasis-related proteins (MMP2 and MMP9) were declined by FXYD3 silence (Figure 1I). Taken together, FXYD3 is notably overexpressed in CC tissues and cells, and FXYD3 up-regulation contributes to the progression of CC.

### FXYD3 is targeted by miR-342-3p

**Figure 2:**
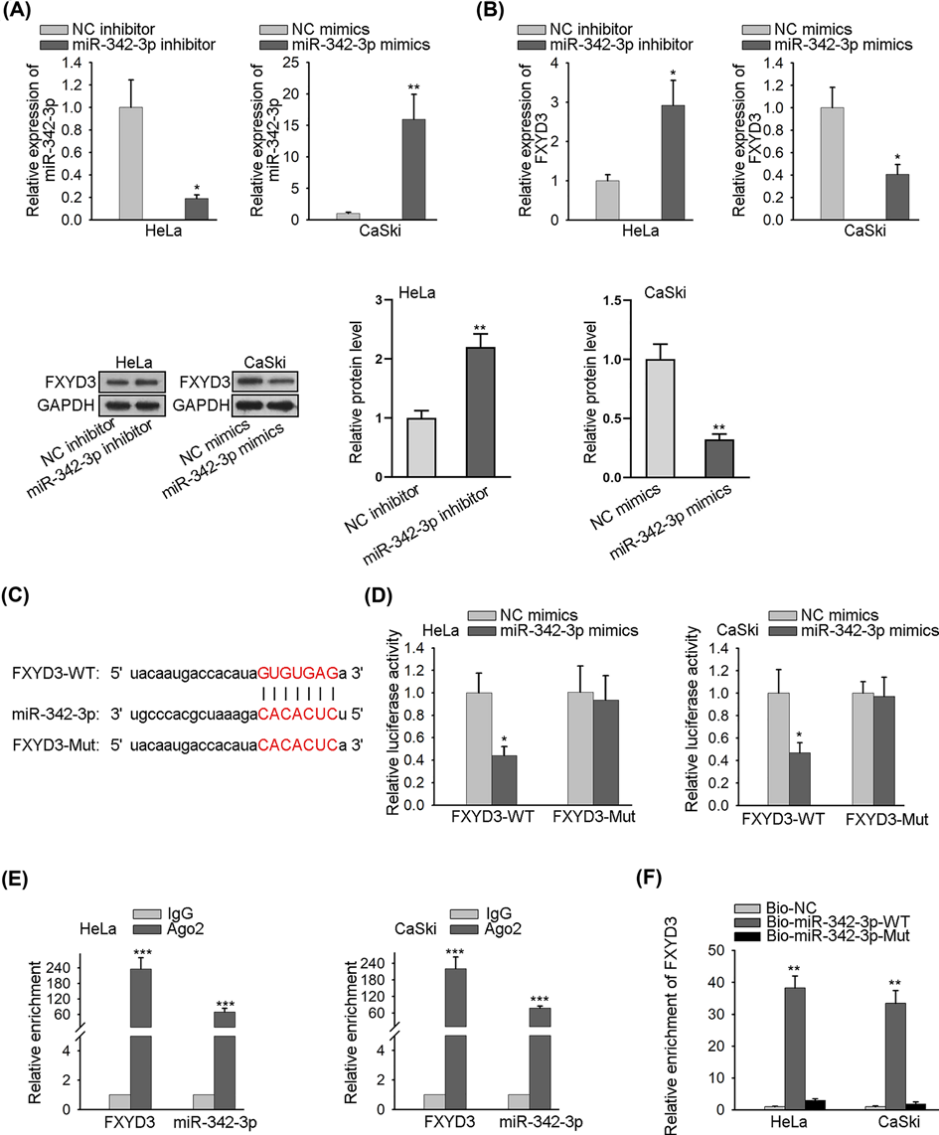
FXYD3 is the target gene of miR-342-3p (**A**) The effect of miR-342-3p mimics or inhibitor on miR-342-3p expression was estimated by RT-qPCR. (**B**) RT-qPCR and Western blot analyses were used to measure FXYD3 mRNA expression and protein level separately in transfected cells. (**C**) The potential miR-342-3p binding site in the sequence of FXYD3 was shown. (**D**) The effect of miR-342-3p mimics on the luciferase activity of FXYD3-WT reporter and FXYD3-Mut reporter was determined by luciferase reporter assay. (**E** and **F**) The interaction between FXYD3 and miR-342-3p was investigated by RIP and RNA pull-down assays; *P<0.05, **P<0.01, ***P<0.001.

Mounting reports have confirmed that lncRNAs serve as a sponge for miRNAs and regulate mRNAs [19]. To explore the upstream gene of FXYD3, we searched Targetscan database (http://www.targetscan.org). And then a series of miRNAs were obtained, of which miR-342-3p was selected due to the highest conservative property. As represented in Figure 2A, miR-342-3p expression was significantly reduced in HeLa cells by transfection with miR-342-3p inhibitor. Meanwhile, miR-342-3p mimics were transfected into CaSki cells for elevating miR-342-3p expression. Subsequently, RT-qPCR and Western blot analyses delineated that the expression levels of FXYD3 mRNA and protein were considerably increased by down-regulated miR-342-3p. In addition, miR-342-3p up-regulation lessened FXYD3 expression (Figure 2B). Then, the binding site between FXYD3 and miR-342-3p was predicted and shown in Figure 2C. To verify the direct interaction between FXYD3 and miR-342-3p, luciferase reporter vectors (FXYD3-WT and FXYD3-Mut) were constructed, and the result of luciferase reporter assay indicated that miR-342-3p mimics tremendously declined the luciferase activity of FXYD3-WT reporter but didn’t affect the luciferase activity of FXYD3-Mut reporter (Figure 2D). RIP assay proved that FXYD3 and miR-342-3p were enriched in the beads conjugated with Ago2 antibody (Figure 2E). Final RNA pull-down assay testified that FXYD3 directly bound to miR-342-3p (Figure 2F). In a word, FXYD3 is targeted by miR-342-3p.

### LINC01503 serves as a sponge for miR-342-3p and positively regulates FXYD3 expression

**Figure 3:**
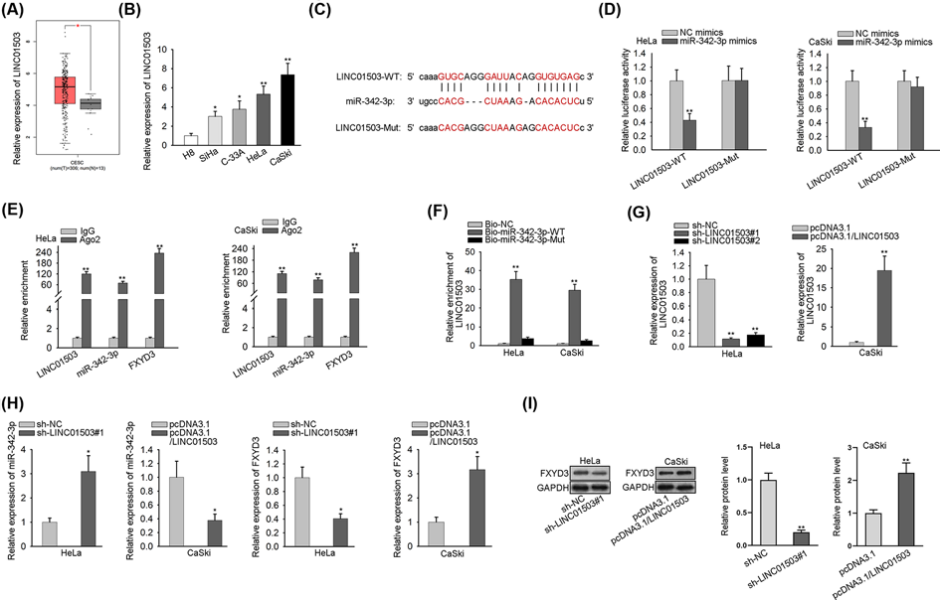
LINC01503 serves as a sponge for miR-342-3p and positively regulates FXYD3 expression (**A**) LINC01503 expression in CC tissues was identified by GEPIA2 database. (**B**) RT-qPCR was conducted to determine LINC01503 expression in CC cell lines. (**C**) The potential miR-342-3p binding site in the sequence of LINC01503 was shown. (**D**) The effect of miR-342-3p mimics on the luciferase activity of LINC01503-WT reporter and LINC01503-Mut reporter was estimated by luciferase reporter assay. (**E** and **F**) The interaction between LINC01503 and miR-342-3p was investigated by RIP and RNA pull-down assays. (**G**) The efficiency of LINC01503 overexpression and knockdown was assessed by RT-qPCR. (**H**) The effect of LINC01503 up-regulation and silence on the expression of miR-342-3p and FXYD3 was tested by RT-qPCR. (**I**) Western blot analysis was applied to detect the effect of LINC01503 overexpression and deficiency on the expression of FXYD3 protein; *P<0.05, **P<0.01.

LncRNAs with the possibility of binding to miR-342-3p were obtained by using the straBase v3.0 website (http://starbase.sysu.edu.cn), and then LINC01503 was screened out because of the highest combining capacity. After online use of GEPIA2 database, we observed that LINC01503 was significantly overexpressed in CC tissues (Figure 3A), which was then further validated in tissue samples collected from CC patients (Supplementary Figure S1D). Additionally, there was a remarkable increase of LINC01503 expression in CC cell lines (Figure 3B). After online search of starBase, the binding site between LINC01503 and miR-342-3p was predicted and illustrated in Figure 3C. And data from luciferase reporter assay demonstrated that miR-342-3p mimics obviously weakened the luciferase activity of LINC01503-WT reporter but not LINC01503-Mut reporter (Figure 3D). After conducting RIP assay, we observed that LINC01503 and miR-342-3p were remarkably enriched by Ago2 antibody (Figure 3E). It was further confirmed by RNA pull-down assay that LINC01503 could combine with miR-342-3p (Figure 3F). Moreover, RT-qPCR analysis revealed that sh-LINC01503#1/2 (or pcDNA3.1/LINC01503) vector resulted in an effective repression (or promotion) in LINC01503 expression, indicated that they could be applied for the subsequent assays (Figure 3G). In addition, interference of LINC01503 elevated miR-342-3p expression whereas lowered FXYD3 expression. Meanwhile, overexpression of LINC01503 exerted an inverted role (Figure 3H). Furthermore, Western blot analysis suggested that the FXYD3 protein level was reduced by LINC01503 depletion, whereas elevated by LINC01503 overexpression (Figure 3I). In brief, LINC01503 sponges miR-342-3p and positively regulates FXYD3 expression.

### LINC01503 aggravates the progression of CC through sponging miR-342-3p to mediate FXYD3 up-regulation

**Figure 4:**
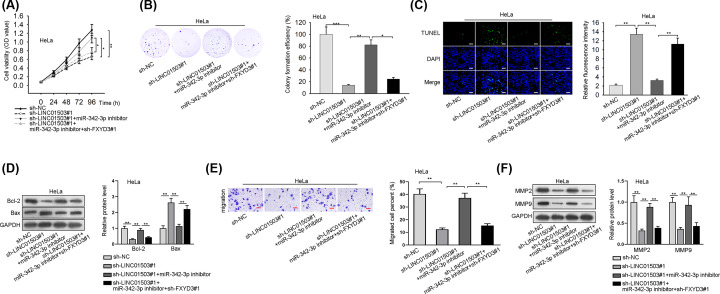
LINC01503 aggravates the tumorigenesis of CC through sponging miR-342-3p to mediate FXYD3 up-regulation (**A** and **B**) The proliferation of transfected cells was tested by CCK-8 and colony formation assays. (**C** and **D**) The apoptosis of transfected cells was analyzed by TUNEL and Western blot; Scale bar: 200 μm, Magnification ×100. (**E** and **F**) Cell migration in different groups was examined via transwell and Western blot; Scale bar: 100 μm, Magnification ×100. *P<0.05, **P<0.01, ***P<0.001.

To investigate whether LINC01503 acted as a regulator for the progression of CC through FXYD3, several rescue experiments were performed. According to the results of CCK-8 and colony formation assays, silenced miR-342-3p counteracted cell proliferation restrained by LINC01503 knockdown, whereas the co-transfection of sh-FXYD3#1 could reverse above phenomenon (Figure 4A,B). TUNEL and Western blot assays uncovered that cell apoptosis promoted in sh-LINC01503#1 group was inhibited by miR-342-3p inhibitor, and then FXYD3 deficiency reversed this inhibitory effect (Figure 4C,D). Further, cell migration ability decreased by LINC01503 knockdown was partially recovered by miR-342-3p inhibitor, and subsequent transfection of sh-FXYD3#1 rescued the effect of sh-LINC01503#1+miR-342-3p inhibitor on the migration of HeLa cells through transwell assay and Western blot analysis (Figure 4E,F). To sum up, LINC01503 aggravates the progression of CC through sponging miR-342-3p to mediate FXYD3 up-regulation.

## Discussion

It has been reported that FXYD3 is closely connected with the progression of various tumors [9,20,21]. However, the role of FXYD3 in CC remains unclear. In the present study, high FXYD3 expression was discovered in CC tissues and cell lines, and low expression of FXYD3 hampered cell proliferation and migration whereas promoted cell apoptosis in CC. Above results revealed that FXYD3 played an oncogenic role in CC.

MicroRNAs (miRNAs) are small RNAs that have no protein-coding capacity and play a key role in cellular activities, such as cell proliferation, migration, invasion and apoptosis [22–24]. Accumulating evidence has clarified that miRNA functions as an oncogene or a tumor-suppressor to modulate mRNA expression in a variety of cancers [25–27]. For instance, miRNA-518 targets MDM2 to inhibit cell growth and induce cell apoptosis in gastric cancer [28]. MiR-200a facilitates EMT process of endometrial cancer via negatively modulating FOXA2 expression [29]. MiR-152-5p functions as an upstream gene of FOXO to restrain cell proliferation and boost cell apoptosis in liver cancer [30]. It has been identified that miR-342-3p act as a tumor suppressor in oral squamous cell carcinoma [31], gastric cancer [32], hepatocellular carcinoma [33] and so on. However, its role in CC has not been elucidated. Our present study depicted that miR-342-3p targeted FXYD3, and negatively modulated FXYD3 expression in CC.

Recent studies have displayed that lncRNAs possibly serve as ceRNAs to modulate mRNAs expression through competitively binding to miRNAs [34]. For example, lncRNA TUG1 interacts with miR-212-3p to enhance cell proliferation and restrain apoptosis by regulating FOXA1 in osteosarcoma [19]. LncRNA Gas5 acts as a sponge for miR-222-3p to activate the progression of papillary thyroid carcinoma by modulating PTEN [35]. SNHG16 induces cell proliferation, migration and EMT process of esophagus cancer through targeting miR-140-5p/ZEB1 axis [36]. LINC01503 has been reported as an oncogene in glioma [6], esophageal squamous cell carcinoma [7] and colorectal cancer [8]. Nonetheless, its underlying role in CC remains largely obscure. Findings in our research manifested that LINC01503 positively regulated FXYD3 expression by competitively binding with miR-342-3p.

In conclusion, LINC01503 facilitates CC progression via miR-342-3p/FXYD3 axis, indicating that LINC01503 can be employed as an underlying diagnostic/therapeutic target for CC.

## Supplementary Material

Supplementary Figure S1

## Abbreviations

CC: cervical cancer
EMT: epithelial-mesenchymal transition
FXYD3: FXYD domain containing ion transport regulator 3
IHC: Immunohistochemistry
lncRNA: long noncoding RNA
