# Supplementary material for: LncRNA LINC01503 aggravates the progression of cervical cancer through sponging miR-342-3p to mediate FXYD3 expression
Source: Biosci Rep. 2020 Jun 10;40(6):BSR20193371. doi: 10.1042/BSR20193371 (PMC7286873; doi:10.1042/BSR20193371)
Supplement: Supplementary Figure S1 [file BSR-2019-3371_supp.pdf]

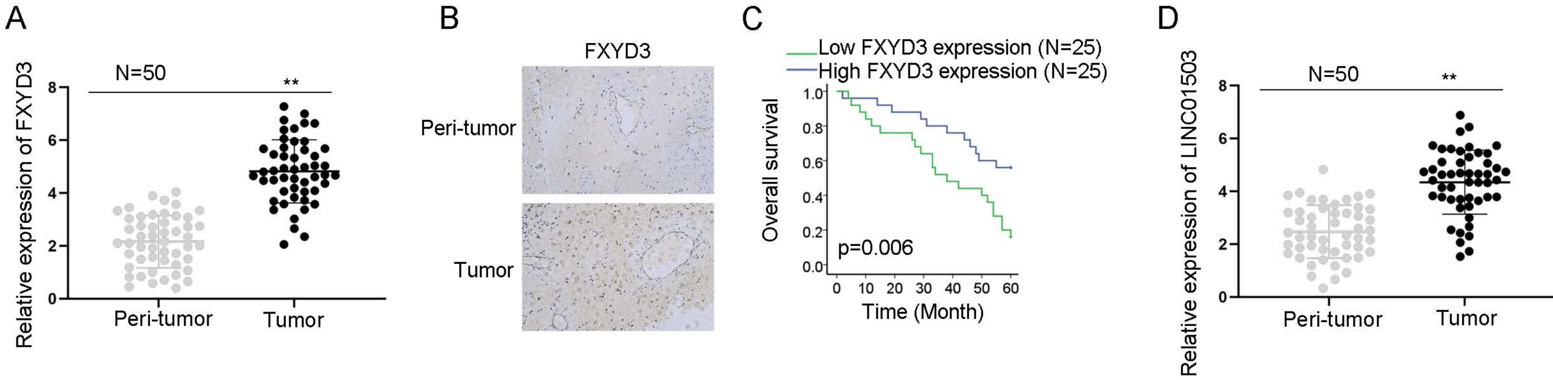

A
N=50
**
Relative expression of FXYD3
8
6
4
2
0
Peri-tumor
Tumor
B
FXYD3
Peri-tumor
Tumor
C
Low FXYD3 expression (N=25)
High FXYD3 expression (N=25)
Overall survival
1.0
0.8
0.6
0.4
0.2
0.0
p=0.006
0 10 20 30 40 50 60
Time (Month)
D
N=50
**
Relative expression of LINC01503
8
6
4
2
0
Peri-tumor
Tumor

Supplementary Figure 1

(A-B)FXYD3expression in CC tissuesandperi-tumor was examined via RT-qPCR and IHC.(C) The association betweenFXYD3expression and overall survival of CC patients was analyzed via Kaplan-Meier analysis. (D)LINC01503expression in CC tissues and peri-tumor was examined via RT-qPCR.$^{**}P < 0.01$.
